# Resistance Reservoirs and Multi-Drug Resistance of Commensal *Escherichia coli* From Excreta and Manure Isolated in Broiler Houses With Different Flooring Designs

**DOI:** 10.3389/fmicb.2019.02633

**Published:** 2019-11-08

**Authors:** Bussarakam Chuppava, Birgit Keller, Amr Abd El-Wahab, Christian Sürie, Christian Visscher

**Affiliations:** ^1^Institute for Animal Nutrition, Foundation University of Veterinary Medicine Hannover, Hanover, Germany; ^2^Department of Nutrition and Nutritional Deficiency Diseases, Faculty of Veterinary Medicine, Mansoura University, Mansoura, Egypt; ^3^Farm for Education and Research Ruthe, Foundation University of Veterinary Medicine Hannover, Ruthe, Germany

**Keywords:** antibiotic resistance, multi-drug resistance, commensal *E. coli*, housing system, flooring design, slatted floor, broilers

## Abstract

Carriage of resistant bacteria and spread of antimicrobial resistance (AMR) in the environment through animal manure pose a potential risk for transferring AMR from poultry and poultry products to the human population. Managing this risk is becoming one of the most important challenges in livestock farming. This study focused on monitoring the prevalence of multi-drug resistance (MDR) bacteria and development of AMR depending on flooring. In two experiments (2 × 15,000 birds), broilers were always divided in two different stables. In the control group, the entire floor pen was covered with litter material and in the experimental group, the flooring system was partly modified by installing elevated slat platforms equipped with water lines and feed pans. Over the whole fattening period, excreta and manure samples were taken (days 2, 22, and 32). In total, 828 commensal *E. coli* isolates were collected. The development and prevalence of resistance against four different antibiotic classes (quinolones, β-lactams, tetracyclines, and sulfonamides) were examined by using broth microdilution. At the end of the trials, the amount of manure per square metre was twice as high below the elevated platforms compared to the control group. Approximately 58% of *E. coli* isolates from excreta showed resistance against at least one antibacterial agent at day 2. During and at the end of the fattening period, resistant *E. coli* isolates at least against one of the four antibacterial agents were observed in excreta (46 and 46%, respectively), and manure samples (14 and 42%, respectively), despite the absence of antibacterial agent usage. In spite of less contact to manure in the experimental group, the prevalence of resistant *E. coli* isolates was significantly higher. Birds preferred the elevated areas which inevitably led to a local high population density. Animal-to-animal contact seems to be more important for spreading antimicrobial resistant bacteria than contact to the litter-excreta mixture. Therefore, attractive areas in poultry housing inducing crowding of animals might foster transmission of AMR. In poultry farming, enrichment is one of the most important aims for future systems. Consequently, there is a need for keeping birds not carrying resistant bacteria at the start of life.

## Introduction

Important resistance problems are the spread of resistant bacteria within and between food-producing animals and humans (Schwarz et al., 2001). Understanding the development as well as transmission of resistant bacteria in intensive poultry production is necessary to implement effective risk management strategies of antibiotic resistance from food-producing animals to humans (GERMAP, 2016; Mehdi et al., 2018; WHO, 2018). Antimicrobial resistance (AMR) is recognised as one of the “One Health” issue that involves links along the potential human-animal-environment infection chain (Aidara-Kane et al., 2018; Collignon and McEwen, 2019). The occurrence of AMR can be caused by horizontal and vertical transmission (Schwarz and Chaslus-Dancla, 2001; McEwen and Collignon, 2018). Multi-drug resistance (MDR) is increasingly reported in poultry worldwide, and due to the impact of these organisms on public health, there is increasing interest in the origin and spread of these organisms in the poultry production chain (Dandachi et al., 2018; Amador et al., 2019).

Antibiotic resistance is a rapidly growing problem in *Enterobacteriaceae*; particularly *E. coli* found in intensive broiler production are developing resistance to multiple antibacterial agents that are important to human health (Hanon et al., 2015; Werckenthin, 2016; Nhung et al., 2017; EFSA and ECDC, 2018). Moreover, it has been reported that the degree of multi-resistance in *E. coli* isolates was highest in broiler chickens compared to other livestock categories (Hanon et al., 2015). *E. coli* are a commensal in the gut microbiome of both humans and animals (Hammerum and Heuer, 2009). Additionally, human and animal gut can be a reservoir of transferable AMR (Rolain, 2013). Commensal *E. coli* are frequently tested for their resistance to antibiotics as they are considered a good indicator of antibiotic exposure of their host (van den Bogaard et al., 2001).

Possible transmission of AMR from poultry products to the human population and additional links to the spread of antimicrobial resistant bacteria in the environment through animal manure have been one of the most important livestock farming challenges for years (van den Bogaard et al., 2001; Kemper, 2009). The poultry industry must develop innovative techniques along the production chain that guarantee high quality and safe consumer products through good management in animal health and welfare as well as in food safety (Windhorst, 2017). Preventive actions to decrease the risk of AMR in order to reduce the need for antimicrobial treatment have to be implemented (De Jong et al., 2014).

Poultry are kept on littered concrete floors for commercial poultry production in Europe (Windhorst, 2017). As a result, the birds spend most of their productive life in close contact with excreta and manure (Kamphues et al., 2011). Thus, these materials can be recognised as a possible reservoir of antibiotic-resistant bacteria (Furtula et al., 2010) and act as a potential reservoir for spreading these organisms to humans via the food chain (Kemper, 2009; OIE, 2018; Amador et al., 2019; Thakur and Gray, 2019).

Information has been reported concerning the effects on the development of AMR in commensal *E. coli* in fattening poultry by separating animals from their excreta under experimental conditions (Chuppava et al., 2018a, b). However, so far, studies have mainly focused on the development of antibiotic-resistant bacteria alongside with antibiotic use (Schwarz and Chaslus-Dancla, 2001; Chantziaras et al., 2013).

The aim of the present study without antibiotic usage was to examine the occurrence and development of AMR and prevalence of multi-drug resistant *E. coli* isolated from excreta and manure samples from large-scale broiler housings with special consideration of the effects of different flooring designs in areas of a stable, which are highly frequented due to their attractiveness.

## Materials and Methods

### Design of Experiment

The broiler chickens in this study were raised under standardised husbandry conditions on the Farm for Education and Research in Ruthe, University of Veterinary Medicine Hannover, Foundation, Germany. The animals were housed in two adjacent buildings, one for the control and one for the experimental groups. The poultry stable was equipped with a gas air-heating system and an automatic temperature, humidity and weight control assembly. The feeding and drinking areas were equipped with a common feed pan system (Big Dutchman International GmbH, Vechta, Germany). A common drinking water system with nipple drinkers for broilers was used (Big Dutchman International GmbH, Vechta, Germany). Dust-free wood shavings were used as litter material and sanitised straw bundles as perching material. Before beginning with the trials, stable floors and all materials had been disinfected as well as screening tests for *Enterobacteriaceae* had been carried out to confirm that they were free of contamination.

Two consecutive trials were conducted with 15,000 birds each. The birds were reared with a stocking density in accordance with German regulations. The experiments started with 1-day-old broiler chickens (as hatched; Ross 308; *N* = 30,000). Broilers had been obtained from a commercial hatchery. The birds in the control and experimental groups each came from the identical hatch for each trial and were randomly distributed to the stables. The growing period lasted about 32 days (d) and was carried out under controlled environmental housing conditions. Animals had *ad libitum* access to fresh, clean water. A commercial pelleted growing diet was offered (MEGA Tierernährung GmbH & Co. KG, Visbek-Rechterfeld, Germany). The feeding programme was divided into three phases. First, a starter feed was offered [12.4 MJ ME/kg, 224 g crude protein (CP)/kg, non-genetically modified organisms (GMO), d 0–11]. From d 12 onward, a grower I followed (12.2 MJ ME/kg, 194 g CP/kg, non-GMO, d 12–19) and after d 20 the feed was changed to a grower II (12.0 MJ ME/kg, 193 g CP/kg, non-GMO, d 20–32). Birds were not given any antibacterial agents throughout the rearing period.

**FIGURE 1:**
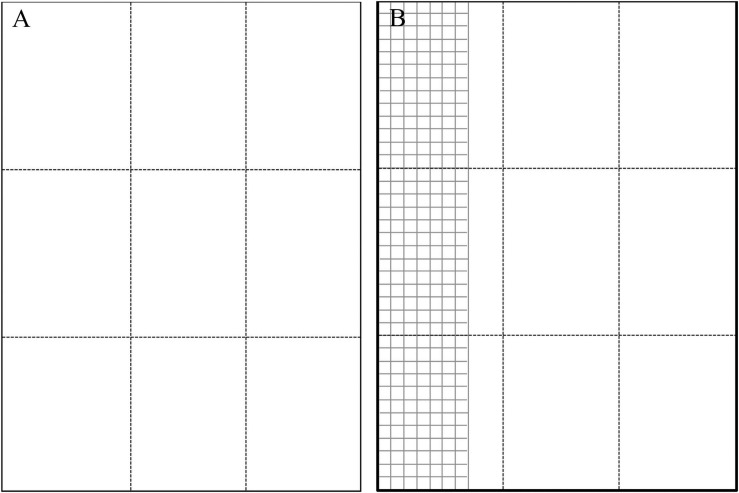
Characteristics of flooring designs: **(A)** Litter (L) = entire floor stable covered with litter (wood shavings); **(B)** Partial-slats (PS) = floor stable with litter and one-quarter slatted flooring. The sample points were distributed over the whole barn area with nine defined places that included drinking areas, feeding areas and the aisle between the drinking and feeding lines.

The first group represented the conventional broiler housing and served as a control. Birds were reared on litter material [Litter (L) – entire stable floor covered with wood shavings; Figure 1A]. In the second group [Partial-slats (PS) – stable floor with wood shavings and partially slatted flooring; Figure 1B], plastic slatted flooring was installed at the west side of the stable (about one-quarter of the total flooring area; legally classified as not belonging to the actual usable flooring area). In this area, there was also the possibility to access feed and water. Plastic slatted-flooring composed of plastic-coated steel slats consisting of holes (15 × 10 mm) and bridges (plastic-covered steel, 3.5 mm wide, Big Dutchman International GmbH, Vechta, Germany). The excreta were stored at a depth of approximately 15 cm below the slatted flooring without removing any material during the entire fattening period. The birds in this study had contact with the litter-excreta mixture during the whole study period in the littered area of the control and the experimental groups.

### Collection of Excreta and Manure Samples

Excreta (*n* = 720) and manure samples (*n* = 108) were collected at three different points of time during the experiment (d2, 22, and 32 of the growing period). Samples were taken from nine defined places on each sampling day (Figure 1). The sample points were distributed over the whole stable area and included drinking areas, feeding areas and the aisle between the drinking and feeding lines. At each area, fresh excreta were sampled from single animals. Manure samples (50 g) were taken with a plastic cup (6 cm in diameter). Material was punched out from the full depth of the litter manure. At the end of each trial, total amounts of manure were replaced, weighed and the dry matter (DM) content was determined so as to calculate the amount of manure per square metre (Supplementary Table S3). Samples were oven-dried over 24 h at 103°C.

### Isolation and Confirmation of *E. col*i

The bacteriological screening was conducted as previously described (Chuppava et al., 2018b). In short: Swab samples with excreta material were processed by streaking on Gassner agar plates (Oxoid Deutschland GmbH, Wesel, Germany), and incubated overnight at 37°C. Twenty-five grammes of each manure sample were suspended in 50 mL of peptone water (Oxoid Deutschland GmbH, Wesel, Germany) and added to a sterile Whirl-Pak^®^ Bag (Nasco International Inc., Fort Atkinson, Wisconsin, United States). The bags were mixed with a Bag Mixer^®^ 400 VW (Interscience International, St Nom la Bretèche, France) for 3 min. Of each mixed-sample, 10 μL was streaked on Gassner agar (Oxoid Deutschland GmbH, Wesel, Germany) and afterward incubated at 37°C for 18–24 h. With the same procedure, one single blue colony from each plate was chosen and spread onto Columbia blood agar (Oxoid Deutschland GmbH, Wesel, Germany) and Tryptone Bile X-glucuronide (TBX) agar (Oxoid Deutschland GmbH, Wesel, Germany). Incubation took place overnight at 37°C. Glucuronidase activity is indicated by blue-green colonies on TBX agar. To confirm the test result, the positive indole test was used with Kovac’s indole reagent (Merck KGaA, Darmstadt, Germany).

### Antibiotic Susceptibility Testing

Minimal inhibitory concentrations (MICs) of enrofloxacin (ENR), ampicillin (AMP), tetracycline (TET) and trimethoprim/sulfamethoxazole (SXT) were examined by using broth microdilution and determined by using Micronaut plates (Merlin^®^, Merlin Diagnostika GmbH, Bornheim-Hersel, Germany) with Mueller-Hinton Broth (Merlin^®^, Merlin Diagnostika GmbH, Bornheim-Hersel, Germany). Dried antibacterial agents in serial dilutions of ENR (0.015625 – 16 μg/mL), AMP (0.125 – 256 μg/mL), TET (0.0078125 – 16 μg/mL), and SXT (0.02/0.30 – 32/608 μg/mL) were placed in wells of these plates. For each isolate, a growth control was added to one well. The manufacturer’s guidelines and those of the Clinical and Laboratory Standards Institute (CLSI) formed the basis for the evaluation of the results. Reference strain, *E. coli* ATCC 25922, was tested concurrently on each testing day.

### Classification Using Clinical Breakpoints

The results were categorised as susceptible (S), intermediate (I) or resistant (R) in accordance with the CLSI veterinary breakpoints available for *Enterobacteriaceae* (CLSI, 2014). The prevalence of antibiotic resistance was defined as the percentage of resistance. MIC distributions for four antibacterial agents were evaluated and summarised as a percentage of frequency. If an isolate with resistance to three or more different antibacterial agents was found, it was defined as MDR (Magiorakos et al., 2012).

### Statistical Analysis

The SAS statistical software package version 7.15 (SAS Inst., Cary, NC, United States) was used to analyse the collected of AMR data. Significant differences were verified among the prevalence of AMR and MDR in isolates acquired from birds from different sampling times by using Pearson’s chi-square test or Fisher’s exact test when expected frequency values were below five. This test was also used to determine the significant differences in the frequency of resistance between the two groups of flooring designs. The statistical significance was set at a *p*-value of *p* < 0.05.

## Results

**FIGURE 2:**
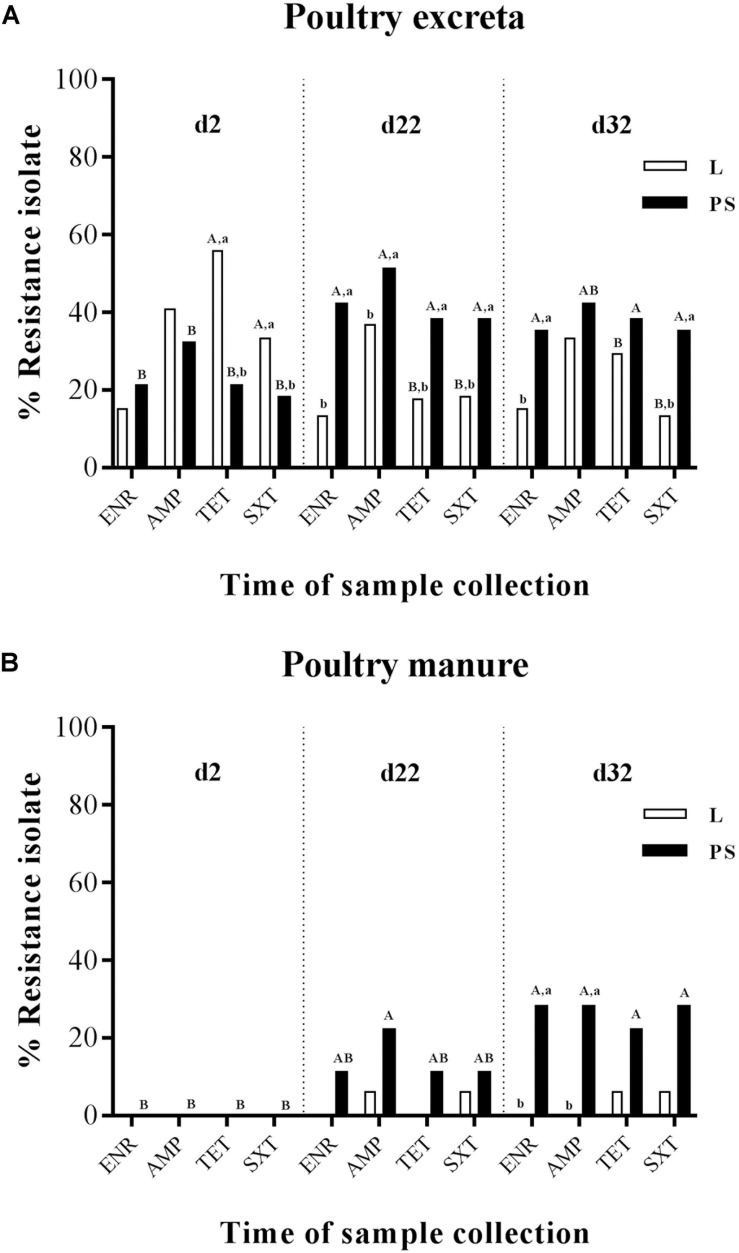
Percentage of frequency of antibiotic resistance of *E. coli* isolates obtained from excreta **(A)** and poultry manure samples **(B)** in different flooring designs [Litter (L) = entire floor stable covered with litter (wood shavings), Partial-slats (PS) = floor stable with litter and one-quarter slatted flooring] at three different sampling times. Relative frequency of resistance patterns exhibited by resistant isolates to four AB. Tested AB: enrofloxacin (ENR), ampicillin (AMP), tetracycline (TET) and trimethoprim/sulfamethoxazole (SXT) in commensal *E. coli* isolates. Excreta samples (*N* = 720; per group d2: *n* = 120, d22: *n* = 120, d32: *n* = 120) and poultry manure samples (*N* = 108; per group d2: *n* = 18, d22: *n* = 18, d32: *n* = 18). Flooring type: □ Litter group (L), ■ Partial-slats group (PS). Different lowercase letters ^(a, b)^ show significant differences in percentage of frequency of antibiotic resistance bacteria depending on flooring (L; PS), specific for time point, separated for each antibiotic (*p* < 0.05). Different upercase letters ^(A, B)^ show significant differences in percentage of frequency of antibiotic resistance bacteria depending on time point specific for flooring (L; PS), separated for each antibiotic (*p* < 0.05).

All 828 *E. coli* isolates (720 excreta samples and 108 manure samples) were tested against four antibiotics (ENR, AMP, TET, and SXT); the results are summarised in Figures 2A,B. The prevalence of resistant *E. coli* was defined as the percentage of excreta (Figure 2A) and manure samples (Figure 2B) for each of the two different flooring designs [Litter (L) and Partial-slats (PS)]. Collection was done at three different times (d2, d22, and d32). Details of the percentage values of resistance to four antibacterial agents in *E. coli* isolates between sampling times and flooring designs are described in Supplementary Tables S1A,B.

### Prevalence of Resistance to Antibacterial Agents in *E. col*i

#### Depending on Sampling Times

When comparing the sampling times (Figures 2A,B; the percentages values of resistant *E. coli* for each collection time are described in detail in Supplementary Tables S1A,B), it became apparent that *E. coli* resistance to ENR, AMP, TET, and SXT isolated from fresh excreta material was already detectable at the beginning of the experiment (d2; Figure 2A). In contrast, at d2, all of the *E. coli* isolates from manure samples were susceptible to all four antibacterial agents (Figure 2B). During the experiment at d22, a significant difference occurred in isolates from the excreta samples (Figure 2A). At the end of the rearing period (d32), the significance between d22 and d32 could not be found in isolates from excreta swabs and manure samples (Figures 2A,B).

Regarding *E. coli* isolated from the fresh excreta material (Figure 2A), the prevalence of resistance to ENR in the PS group significantly increased from the start to the middle of the experiment at d22 (21–42%, respectively; Supplementary Table S1A). Nevertheless, the ENR-resistant *E. coli* isolates from the excreta samples (Figure 2A) and manure samples (Figure 2B) demonstrated no significant differences in resistance between d22 and d32.

Ampicillin-resistant *E. coli* isolates were detected from fresh excreta swabs (Figure 2A) at the start of fattening. The highest tested prevalence of resistance to AMP was detected in excreta swab and manure samples at d22 in the PS group (51 and 22%, respectively). In addition, the percentage of samples with isolation of resistant *E. coli* among excreta swabs and manure samples significantly increased from d2 to d22; 32% to 51% and 0% to 22%, respectively (Figures 2A,B).

The results of percentage of tetracycline-resistant *E. coli* isolates from fresh excreta swabs showed a significant decrease from the beginning to the middle of the experiment in group L (56% to 18%; Figure 2A). On the contrary, the percentage of TET-resistant *E. coli* isolates in the PS group increased from 21% to 38% during the experiment (Figure 2A).

Significant differences between the sampling times could also be found in trimethoprim/sulfamethoxazole-resistant *E. coli* isolates (Figures 2A,B). The prevalence of resistance in fresh excreta swabs significantly decreased from the beginning to the middle of the experiment in group L (33% to 18%; Figure 2A). On the other hand, in the PS group, the percentage of excreta samples with isolation of resistant *E. coli* significantly increased from d2 to d22 (18% to 38%; Figure 2A). In manure samples, there were no significant differences between d2 and d22 (Figure 2B).

#### Depending on Flooring Designs

At the beginning of the experiment (d2), there was no significant difference in percentage of isolates from excreta resistant against ENR and AMP between the L and PS groups, whereas isolates where more often resistant against TET and SXT in the L group at start (Figure 2A). TET-resistant *E. coli* isolates showed the significantly highest prevalence (Supplementary Table S1A). There were significant differences between flooring systems in this collection time. The prevalence of TET-resistant isolates was significantly different between the L and PS groups, 56 and 21%, respectively (*p*-value < 0.05; Figure 2A and Supplementary Table S1A). At the same point in time, SXT-resistant isolates in the L group also showed significantly higher resistance rates compared with the isolates collected from the PS group, 33 and 18%, respectively (*p*-value < 0.05; Figure 2A and Supplementary Table S1A). On the other hand, none of the *E. coli* isolates from manure samples showed any differences between the groups at d2 (Figure 2B). The resistance rates in the L and PS groups were similar.

During the experimental period (d22), a significant difference between the two flooring systems was found only in isolates from fresh excreta swabs (Figure 2A). At this point, resistant *E. coli* isolates of all four antibacterial agents in the L group showed significantly lower percentages than in the PS group; ENR: 13 and 42%, AMP: 37 and 51%, TET: 18 and 38% and SXT: 18 and 38%, respectively (*p*-value < 0.05; Supplementary Table S1A). For manure samples, there were no significant differences in the resistance between the L and PS groups during this sampling time (Figure 2B).

At the end of the fattening period (d32), the *E. coli* isolates in L group also showed significantly lower percentages in resistance to ENR and SXT acquired from fresh excreta swabs than in the PS group (ENR: 15 and 35% and SXT: 13 and 35%, respectively; Figure 2A and Supplementary Table S1A). However, the resistant *E. coli* isolates acquired from manure samples (Figure 2B and Supplementary Table S1B) at d32 showed significant differences between the L and PS groups (ENR: 0 and 28% and AMP: 0 and 28%, respectively; *p*-value < 0.05). Regarding TET and SXT resistance in isolates from the manure samples (Figure 2B), there were no significant differences between the groups.

### MIC Distribution of the Commensal *E. coli* Isolates

**FIGURE 3:**
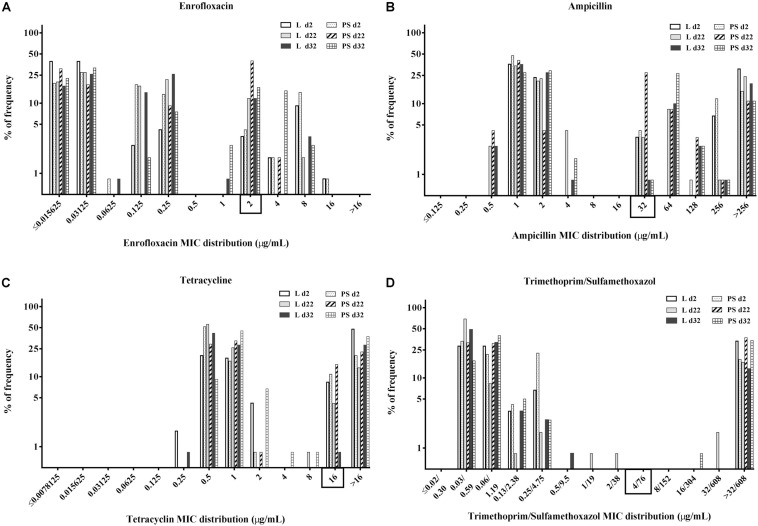
Percentage of frequency of minimum inhibitory concentration (MIC) distribution of enrofloxacin **(A)**, ampicillin **(B)**, tetracycline **(C),** and trimethoprim/sulfamethoxazole **(D)** in commensal *E. coli* isolates from excreta samples (*N* = 720; per group d2: *n* = 120, d22: *n* = 120, d32: *n* = 120). Rectangle on the *x*-axis: Categorisation by using CLSI breakpoints for *Enterobacteriaceae*, enrofloxacin (ENR) ≥ 2 μg/mL, ampicillin (AMP) ≥ 32 μg/mL, tetracycline (TET) ≥ 16 μg/mL and trimethoprim/sulfamethoxazole (SXT) ≥ 4/76 μg/mL. Flooring type: Litter (L) = entire floor stable covered with litter (wood shavings); Partial-slats (PS) = floor stable with litter and one-quarter slatted flooring.

Minimal inhibitory concentration distribution of the 720 commensal *E. coli* isolates (percentage of frequency) from fresh excreta was tabulated separately for each antibacterial agent depending on sampling time points and flooring design according to age (two, 22 and 32 days of age, respectively) in Figures 3A–D. Categorisation was done by using CLSI breakpoints [ENR ≥ 2 μg/mL, AMP ≥ 32 μg/mL, TET ≥ 16 μg/mL and SXT ≥ 4/76 μg/mL] for *Enterobacteriaceae* (CLSI, 2014).

When isolates were compared based on the flooring types in the two groups, the overall percentages of resistant *E. coli* isolates in the PS group were higher than those in the L group (Figures 3A–D). When isolates were grouped according to time of collection, the overall resistance percentages were higher at d22 compared with the others sampling times (Figures 3A–D).

Regarding the MICs distribution for ENR (Figure 3A), a high percentage of resistant *E. coli* isolates was found at d22 in the PS group, with 42% isolates (*n* = 120) having enrofloxacin MICs of 2 μg/mL. Approximately 37 and 21% of isolates from fresh excreta samples (*N* = 720) had ampicillin MICs of 1 and 2 μg/mL, respectively (Figure 3B), with 18% of isolates (*N* = 720) having AMP MICs of >256 μg/mL. Concerning the percentage of isolates with tetracycline MICs (Figure 3C), approximately 66% of *E. coli* isolates from fresh excreta samples (*N* = 720) had MIC-values below the clinical breakpoint (MIC < 16 μg/mL). Approximately 26% of *E. coli* isolates from fresh excreta samples (*N* = 720) had SXT MIC-values above the clinical breakpoint (MIC ≥ 4/76 μg/mL; Figure 3D).

### Prevalence of Multi-Drug Resistance and Resistance Pattern

**FIGURE 4:**
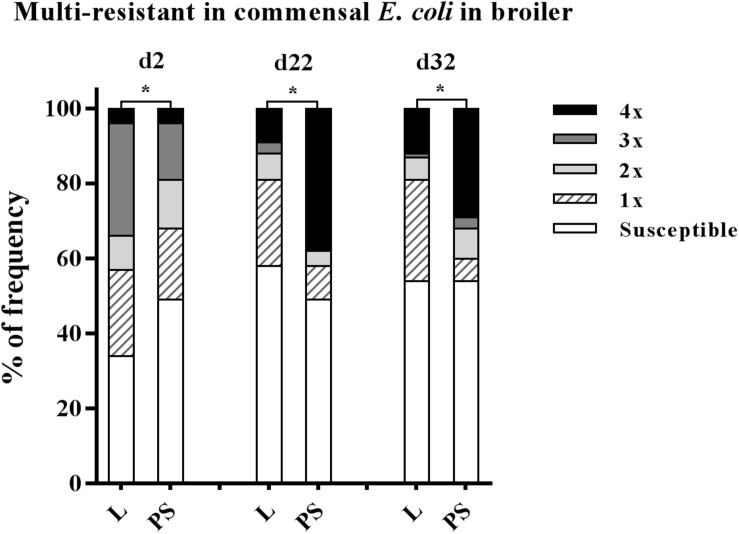
Prevalence of multi-resistant commensal *E. coli* isolates between the flooring designs. Litter (L) = entire floor stable covered with litter (wood shavings), Partial-slats (PS) = floor stable with litter and one-third slatted flooring at three sampling times. Multi-resistance was considered as resistance by an isolate to at least three antimicrobials belonging to different antimicrobial classes. Excreta samples (*N* = 720; per group d2: *n* = 120, d22: *n* = 120, d32: *n* = 120). Percentage of multi-resistance between L and PS groups differ significantly (^∗^*p* < 0.0001).

Isolate resistance to at least three antibacterial agents was defined as MDR (Magiorakos et al., 2012), belonging to different antibiotic classes: enrofloxacin (fluoroquinolones (FQ) class), ampicillin (β-lactams class), tetracycline (tetracyclines class), trimethoprim/sulfamethoxazole (sulphonamides class). The values of multi-resistance in *E. coli* for each collection time and group are given in detail in Supplementary Table S2. At the beginning of the study, the MDR rates were significantly higher in L group samples (34 and 19% in L and PS group, respectively; *p*-value < 0.001; Figure 4). The highest levels of MDR were found at d 22 in the PS group (38%; Figure 4). The prevalence of MDR continually increased in *E. coli* isolates in the PS group from d2 to d22 (19% to 38%; Supplementary Table S2).

Overall prevalence of AMR in the present study showed a high percentage of *E. coli* isolates resistant to at least one antibiotic agent from day-old chicks’ excreta in both the L and PS groups, 66 and 51%, respectively, (Supplementary Table S2) as well as 33% isolated from the paper inlays from the transport boxes (data not shown). During and at the end of the fattening period (Supplementary Table S2), *E. coli* isolates resistant to at least one antibiotic agent were observed in fresh excreta samples in both the L and PS groups (d22: 42 and 51%; d32: 46 and 46%, respectively), despite the absence of antibacterial agent usage in this study.

The 720 resistant *E. coli* isolates from fresh excreta samples in this study were grouped into fourteen different resistance patterns (Table 1). Approximately fifty percent of all isolates from these swabs (359 out of 720) show resistance to at least one of the tested antibacterial agents (Table 1), 19% were individually resistant (130 out of 720) and of these 130 isolates, 64 of them showed resistance to AMP. Seven per cent were resistant to two antibacterial agents and 24% (176 out of 720) were defined as multi-resistant (having at least three resistance determinants). The most common resistance patterns in this study were ENR-AMP-TET-SXT (119 isolates out of 720). The highest number of broad-spectrum resistance pattern (ENR-AMP-TET-SXT) was found at d22 in isolates from the PS group (45 isolates; Table 1). Regarding other additional resistance patterns, the prevalence of MDR was observed in excreta samples (*N* = 720; Table 1); AMP-TET-SXT (56 isolates), AMP-TET (26 isolates), ENR-AMP (11 isolates), ENR-TET (eight isolates), TET-SXT (six isolates), AMP-SXT (two isolates), and ENR-AMP-SXT (one isolate).

**TABLE 1:** Antibiotic resistance pattern for *E. coli* isolates obtained from excreta samples (*N* = 720) in different flooring designs at the age of 2, 22, and 32 days.

**No. antibacterial agents**	**Antibiotic resistance pattern^∗^**	**Number of isolates**						
		**d2 (*n* = 240)**		**d22 (*n* = 240)**		**d32 (*n* = 240)**		**Total**
		**L**	**PS**	**L**	**Ps**	**L**	**PS**	
	Susceptible to all	41	60	71	59	66	64	361
1	ENR	12	17	1	0	2	1	33
	AMP	0	7	25	11	16	5	64
	TET	16	3	0	0	13	1	33
	SXT	0	0	0	0	0	0	0
2	ENR-AMP	0	1	0	5	1	4	11
	ENR-TET	5	2	1	0	0	0	8
	ENR-SXT	0	0	0	0	0	0	0
	AMP-TET	9	8	0	0	6	3	26
	AMP-SXT	0	0	2	0	0	0	2
	TET-SXT	0	0	3	0	0	3	6
3	ENR-AMP-TET	0	0	0	0	0	0	0
	ENR-AMP-SXT	0	0	0	0	0	1	1
	AMP-TET-SXT	33	17	3	0	1	2	56
4	ENR-AMP-TET-SXT	4	5	14	45	15	36	119

*^∗^ENR, enrofloxacin; AMP, ampicillin; TET, tetracycline; SXT, trimethoprim/sulfamethoxazole.*

## Discussion

Antibiotic resistance is a global health threat (WHO, 2018). Concerns due to the emergence of AMR in humans are justified by the occurrence of AMR in animals and their environment (Schroeter and Kaesbohrer, 2010; DART, 2015). AMR in poultry production is one of its contributing sources. This has been the major topic of a large number of studies in recent years on single drug resistance as well as MDR in poultry (Furtula et al., 2013; Diarra and Malouin, 2014; Nhung et al., 2017; Chuppava et al., 2018a; McEwen and Collignon, 2018; Roth et al., 2018; Thakur and Gray, 2019).

### Resistance to Antibacterial Agents Found in Day-Old Chicks

*E. coli* isolated from 2-day-old chicks’ excreta in the present study showed resistance to enrofloxacin, ampicillin, tetracycline and trimethoprim/sulfamethoxazole, despite the birds not having been previously in contact with antibacterial agents as well as stable floors and all materials were free of *Enterobacteriaceae* contamination before beginning with the trials. Similar results were reported in previous studies (Jiménez-Belenguer et al., 2016; Chuppava et al., 2018a, b) finding *E. coli* from 1-day-old chicks resistant to ENR, AMP, TET and SXT; and also from chicks on laying hen farms (Moreno et al., 2019).

The high resistance rates found in our study including from the paper inlays from the transport boxes, could be associated with vertical transmission of resistant isolates from parent flocks as described in literature (Petersen et al., 2006; ECDC et al., 2017). Contamination during incubation in the hatchery or during transport seems to be the most probable explanation (Dierikx et al., 2013; Projahn et al., 2018). Hatchery-related factors can generally influence the occurrence of resistance to antibacterial agents (Persoons et al., 2011). In newly hatched chicks, the bacteria found in the environment, whether they are susceptible or resistant, colonise the gut and become part of the normal intestinal flora (Persoons et al., 2011). Therefore, the possible explanation for the resistance rates found in our study could be due to contamination of chickens by vertical transmission.

According to previous findings (Petersen et al., 2006; Bortolaia et al., 2010; Moreno et al., 2019), resistance to FQ, β-lactams, tetracycline and sulphonamides in *E. coli* was related to parent chickens. Therefore, it is possible to have vertical transmission of AMR of commensal *E coli* that were selected for AMR long ago and remain as commensal populations within the hatchery or in stable, whether acquired resistance or natural. However, in every single case, other possible sources of contamination such as the antibiotic usage upstream the hatchery cannot be ruled out.

The findings regarding young animals as a potential reservoir of AMR in this study suggest that besides the effects of the management practices known from previous studies, the focus of reduction approaches should be on implementing poultry hatcheries and sources along the distribution chain to control the spread of AMR. Consequently, further research is strongly recommended, paying particular attention to analysing the genetic basis of resistance in the isolates. This should be done on as many isolates as possible to avoid bias from sample selection. This should be done in order to understand the origin, development as well as transfer of the resistance mechanism.

### Broiler Chickens Excreta and Manure Harboured Antibiotic Resistant *E. col*i

The occurrence of resistant and multi-resistant *E. coli* isolates was shown in this study. The observed high prevalence of resistance in *E. coli* to four antibacterial agents in isolates from excreta and manure, particularly during the fattening period, may be a consequence of intensive animal farming (Nhung et al., 2017). Therefore, it has been hypothesised that animals as well as manure may be the reservoir of these resistant bacteria. Similar findings of prevalence of *E. coli* isolates from broilers and their products, particularly meat, resistant to FQ, β-lactam, tetracyclines, and sulfonamides were frequently found in other studies (Furtula et al., 2010; Ozaki et al., 2011; Rugumisa et al., 2016; EFSA and ECDC, 2018). Regarding development of AMR over time, our data show increasing trend, unlike other studies (Diarra et al., 2007). Diarra et al. (2007) had an insignificantly decreasing trend in the occurrence of AMR noted in *E. coli* isolates as the birds aged.

A relatively high prevalence of *E. coli* isolates (50%) was found to be resistant to multiple antibiotics. Interestingly, approximately 17% of *E. coli* isolates showed a resistance pattern to four antibiotics (ENR-AMP-TET-SXT), in spite of antibiotics not having been used in our study. Our findings agree with previous reports from Germany (GERMAP, 2016), from European countries (Chaslus-Dancla et al., 1987; Amador et al., 2019) and global ones (Nhung et al., 2017) that found resistance in *E. coli* isolated from poultry farms to many classes of antibiotics, including FQ, β-lactams, tetracyclines and sulfonamides. Similarly, Ozaki et al. (2011) reported that the amount of resistance in *E. coli* detected among isolates from excreta increased during the growth of chickens. Therefore, it was not possible to determine where the resistant bacteria originated from even in the absence of antibiotic administration. However, our study demonstrates that these bacteria carry out to other animals in the same stable despite the absence of selection pressure related to the non-use of antimicrobial agents. Due to the increasing prevalence of resistant bacteria during fattening, one can assume that a transfer of resistances or resistant bacteria did occurred in this study.

Similarities among these antibiotics (Poirel et al., 2018) show a need for further studies to analyse whether these resistances may have developed during the growing phase or whether the explanation for the difference in terms of prevalence of AMR in each sampling time is possibly due to intensive animal-to-animal contact transmission. Horizontal transmission greatly contributes to the widespread dissemination of AMR (Khan et al., 2005). In our study, at the beginning of the experiment birds carried resistant bacteria. These bacteria might be spread from the digestive tracts via shedding and persist in the environment (Diarra and Malouin, 2014). This could result in rapid contamination of the other individuals in the same flock and in the stable environment (Jiménez-Belenguer et al., 2016). Nevertheless, the role of contamination of the animal’s direct environment through dust in the stable should be taken into account. Dust formation (litter, feather/skin particles, excreta, etcetera) is common in practice, so that a possible particle-related transmission of resistant bacteria between animals could not be ruled out because particles could also be transported by air (Schulz et al., 2016).

There are various possible explanations for an increase or a decrease in antibiotic resistance in *E. coli*. More importantly, however, it constitutes a major and shared reservoir of resistance determinants to most families of antibacterial agents transmitted by animals. Despite this, the different transmission pathways of resistant *E. coli* isolates in this study remain to be clarified. The transmission may include direct contact with animals or indirect transfer through the environment. As no further genetic analyses were carried out, the reason for this difference remains unknown. Development of resistance is very complex. We cannot regard all interactions when we only obtain one isolate from a sample and then by way of example, try to conclude the complexity of resistance development. Therefore, more research is required to find possible explanations concerning the mechanism behind the shedding of antibacterial agent resistant *E. coli* in broilers.

### Relationship Between Flooring System and the Occurrence of Resistance in the Isolates

Few studies have examined reducing the development of resistance to antibacterial agents by applying different flooring designs simulating different contact intensity between animals and their manure. Despite the fact that the prevalence of resistant *E. coli* depending on flooring design has been documented (Chuppava et al., 2018a,b), information is lacking concerning the MDR pattern to antibacterial agents in large-scale broiler farming.

Wright (2010) and Furtula et al. (2010) stated that the environment, including dirty litter, could also be a potential reservoir of resistant bacteria. Nevertheless, our study in the PS group, the animals had less contact to litter material, the development of AMR still occurred in these animals, or rather, it was even more protracted. Not only the prevalence of resistant *E. coli* isolates from excreta material was significantly higher, but also the highest percentage of multi drug-resistant *E. coli* was found in the partially slatted flooring group (PS) compared to the litter group during the experimental period. Interestingly, the results were contrary to our expectations, as significantly higher AMR was shown in the PS group where the animals in about one-quarter of the stable had no direct contact to the excreta because of using elevated levels with slatted flooring. In contrast, Chuppava et al. (2018b) concluded from their experimental model that a lower exposure to resistant bacteria in manure might lead to a lower percentage of resistant *E. coli* isolates in their study animals. However, their research was an experimental study with very small animal groups in which the effects of crowding were irrelevant. Therefore, additional focus is needed elsewhere.

The animals in the PS group in our study preferred the elevated areas (areas with the possibility of simultaneous intake of water and feed) and thus, a high population density in these areas was observed. The amount of manure in the respective areas allows this conclusion to be drawn. Below the slatted areas, the amount of manure per square metre was higher than in the littered area of the same stable or in the control stable in general. While between 12.6 and 13.3 kg (on DM-basis) of litter per square metre was found in the control group housing, up to 24.2 kg was found below the slats (Supplementary Table S3). High stocking density is common is poultry farming. Crowding can be an important factor inducing AMR in bacteria. This may be an explanation for the high prevalence and multi-resistance in faecal *E. coli* of poultry in this and other studies (van den Bogaard et al., 2001; Nhung et al., 2017).

When analysing the results from the final litter quality and litter moisture content in the poultry manure between the L and PS groups (Supplementary Table S3), the quality of the manure in the PS group was characterised by the lowest values of DM content (highest moisture content with loose structure) in the slatted flooring area, compared with the other areas in the same stable. Although there was no direct contact with the animals, this may have been a good reservoir for viable bacteria. Further studies should therefore clarify whether this directly affects the development of AMR or whether the higher temporary animal density described above is relevant. The first question could be clarified by permanently removing the excrements below the elevated levels.

## Conclusion

According to our findings, animals carrying resistance at the start of the fattening period can be a reservoir and the starting point for the transmission of bacterial resistance to the other animals in the same flock. Elevated areas, particularly if there is a possibility of accessing feed and drinking water, seem to be very attractive, which is reflected in high amounts of manure beneath these areas. This can induce crowding of animals. The resulting animal-to-animal contact seems to be more important for spreading of resistance than contact to the litter material. This might foster transmission of AMR within the whole flock. To resolve the problems with AMR, one important requirement is still that of obtaining animals not carrying resistance at the start of life. The absence of resistance is all the more important the more the environment of the animals leads to an intensive animal-to-animal contact (high density overall, attractive stable areas with partially high animal density, etcetera).

## Data Availability Statement

The datasets generated for this study are available on request to the corresponding author.

## Ethics Statement

The University of Veterinary Medicine Hannover, Foundation, has an Animal Protection Office. This is the local committee dealing with ethical questions regarding animal experiments. The experiments were carried out in accordance with German regulations (Animal Protection Act). No direct interventions were carried out on animals which could be associated with pain, suffering or damage to these animals. Solely excreta samples and litter material were tested for antimicrobial resistance. This sample material was collected during common fattening of broiler chickens. For this reason, these examinations required no announcement or permission with regard to the animal protection law (§7, paragraph 2) since no measures inflicting pain, suffering or damage on these animals were carried out.

## Author Contributions

CV and CS conceptualised the study and acquired funding. CV and BK designed the methodology. BC performed the experiments. BC and AA collected the samples. BC and CV analysed the data. The original manuscript draft was prepared by BC and CV. CV reviewed and edited the manuscript and supervised the project. All authors contributed to reading the manuscript and approving the submitted version.

## Conflict of Interest

The authors declare that the research was conducted in the absence of any commercial or financial relationships that could be construed as a potential conflict of interest.
