# Supplementary material for: Resistance Reservoirs and Multi-Drug Resistance of Commensal Escherichia coli From Excreta and Manure Isolated in Broiler Houses With Different Flooring Designs
Source: Front Microbiol. 2019 Nov 8;10:2633. doi: 10.3389/fmicb.2019.02633 (PMC6857331; doi:10.3389/fmicb.2019.02633)
Supplement: Supplementary file 1 [file Table_1.docx]

## Supplementary Material

**SUPPLEMENTARY TABLE S1A** Percentage of frequency of antibiotic resistance in commensal *E. coli* isolates obtained from poultry excreta samples in different flooring designs at three different sampling times.

| % resistance |  | ENR^*^ | |  | AMP^*^ | |  | TET^*^ | |  | SXT^*^ | |
| --- | --- | --- | --- | --- | --- | --- | --- | --- | --- | --- | --- | --- |
|  |  | L | PS |  | L | PS |  | L | PS |  | L | PS |
| d2 |  | 15^A,a^ | 21^B,a^ |  | 41^A,a^ | 32^B,a^ |  | 56^A,a^ | 21^B,b^ |  | 33^A,a^ | 18^B,b^ |
| d22 |  | 13^A,b^ | 42^A,a^ |  | 37^A,b^ | 51^A,a^ |  | 18^B,b^ | 38^A,a^ |  | 18^B,b^ | 38^A,a^ |
| d32 |  | 15^A,b^ | 35^A,a^ |  | 33^A,a^ | 42^AB,a^ |  | 29^B,a^ | 38^A,a^ |  | 13^B,b^ | 35^A,a^ |

^A, B^ means in the same column differ signiﬁcantly between sampling stages (p<0.05);
^a, b^ means differ signiﬁcantly between the group (p<0.05); excreta samples: N = 720; per group d2: n = 120, d22: n = 120, d32: n = 120. Litter (L) = entire ﬂoor stable covered with litter; partial-slats (PS) = one-fourth slatted ﬂooring including a littered area.

* ENR, enrofloxacin; AMP, ampicillin; TET, tetracycline; SXT, trimethoprim/sulfamethoxazole.

**SUPPLEMENTARY TABLE S1B** Percentage of frequency of antibiotic resistance in commensal *E. coli* isolates obtained from poultry manure samples in different flooring designs at three different sampling times.

| % resistance | |  | ENR^*^ | | |  | AMP^*^ | |  | TET^*^ | |  | SXT^*^ | |
| --- | --- | --- | --- | --- | --- | --- | --- | --- | --- | --- | --- | --- | --- | --- |
|  |  |  | L | | PS |  | L | PS |  | L | PS |  | L | PS |
| d2 |  | | | 0^A,a^ | 0^B,a^ |  | 0^A,a^ | 0^B,a^ |  | 0^A,a^ | 0^B,a^ |  | 0^A,a^ | 0^B,a^ |
| d22 |  | | | 0^A,a^ | 11^AB,a^ |  | 6^A,a^ | 22^A,a^ |  | 0^A,a^ | 11^AB,a^ |  | 6^A,a^ | 11^AB,a^ |
| d32 |  | | | 0^A,b^ | 28^A,a^ |  | 0^A,b^ | 28^A,a^ |  | 6^A,a^ | 22^A,a^ |  | 6^A,a^ | 28^A,a^ |

^A, B^ means in the same column differ signiﬁcantly between sampling stages (p<0.05);
^a, b^ means differ signiﬁcantly between the group (p<0.05); poultry manure samples: N = 108; per group d2: n = 18, d22: n = 18, d32: n = 18. Litter (L) = entire ﬂoor stable covered with litter; partial-slats (PS) = one-fourth slatted ﬂooring including a littered area.

* ENR, enrofloxacin; AMP, ampicillin; TET, tetracycline; SXT, trimethoprim/sulfamethoxazole.

**SUPPLEMENTARY TABLE S2** Prevalence of multi-resistant commensal *E. coli* isolates, between the flooring designs at three sampling stages.

| % resistance |  | No. of antibacterial agent resistance | | | | | | | | | | | | | |
| --- | --- | --- | --- | --- | --- | --- | --- | --- | --- | --- | --- | --- | --- | --- | --- |
|  |  | Susceptible | |  | 1 | |  | 2 | |  | 3 | |  | 4 | |
|  |  | L | PS |  | L | PS |  | L | PS |  | L | PS |  | L | PS |
| d2 |  | 34^B,a^ | 49^A,a^ |  | 23^A,b^ | 19^B,a^ |  | 9.0^A,a^ | 13^A,a^ |  | 30^A,a^ | 15^A,b^ |  | 4.0^A,a^ | 4.0^B,a^ |
| d22 |  | 58^A,a^ | 49^A,a^ |  | 23^A,a^ | 9^B,b^ |  | 7.0^A,a^ | 4.0^A,a^ |  | 3.0^B,a^ | 0.0^B,a^ |  | 9.0^A,b^ | 38^A,a^ |
| d32 |  | 54^A,a^ | 54^A,a^ |  | 27^A,a^ | 6.0^B,b^ |  | 6.0^A,a^ | 8.0^A,a^ |  | 1.0^B,a^ | 3.0^B,a^ |  | 12^A,b^ | 29^AB,a^ |

^A, B^ means in the same column differ signiﬁcantly between sampling stages (p<0.05);
^a, b^ means differ signiﬁcantly between the group (p<0.05); excreta samples: N = 720; per group d2: n = 120, d22: n = 120, d32: n = 120. Litter (L) = entire ﬂoor pen covered with litter; partial-slats (PS) = partially slatted ﬂooring including a littered area. Multi-drug resistance (MDR) was considered as resistance by an isolate to at least three antimicrobials belonging to different antimicrobial classes.

**SUPPLEMENTARY TABLE S3** Litter quality and litter quantity per square metre when comparing common housing systems without (Litter) and with elevated areas (Partial-slats) in two trials.

|  |  |  | Litter | Partial-slats | |
| --- | --- | --- | --- | --- | --- |
|  |  |  |  | Slatted | Litter |
| Dry matter content litter | [%] | Trial 1 | 65.3 | 60.4 | 72.2 |
|  |  | Trial 2 | 58.3 | 52.6 | 67.3 |
| Litter / m^2^ | kg / m^2^ | Trial 1 | 13.3 | 24.2 | 8.43 |
|  |  | Trial 2 | 12.6 | 16.8 | 9.08 |
